# A Systematic Review of the Associations Between Inverse Dynamics and Musculoskeletal Modeling to Investigate Joint Loading in a Clinical Environment

**DOI:** 10.3389/fbioe.2020.603907

**Published:** 2020-12-07

**Authors:** Jana Holder, Ursula Trinler, Andrea Meurer, Felix Stief

**Affiliations:** ^1^Faculty of Medicine, Goethe University Frankfurt, Frankfurt am Main, Germany; ^2^Movement Analysis Laboratory, Orthopedic University Hospital Friedrichsheim gGmbH, Frankfurt am Main, Germany; ^3^Laboratory for Movement Analysis, BG Trauma Center Ludwigshafen, Ludwigshafen, Germany; ^4^Department of Special Orthopedics, Orthopedic University Hospital Friedrichsheim gGmbH, Goethe University Frankfurt, Frankfurt am Main, Germany

**Keywords:** musculoskeletal modeling, inverse dynamics, gait analysis, joint contact forces, joint moments, knee joint, hip joint

## Abstract

The assessment of knee or hip joint loading by external joint moments is mainly used to draw conclusions on clinical decision making. However, the correlation between internal and external loads has not been systematically analyzed. This systematic review aims, therefore, to clarify the relationship between external and internal joint loading measures during gait. A systematic database search was performed to identify appropriate studies for inclusion. In total, 4,554 articles were identified, while 17 articles were finally included in data extraction. External joint loading parameters were calculated using the inverse dynamics approach and internal joint loading parameters by musculoskeletal modeling or instrumented prosthesis. It was found that the medial and total knee joint contact forces as well as hip joint contact forces in the first half of stance can be well predicted using external joint moments in the frontal plane, which is further improved by including the sagittal joint moment. Worse correlations were found for the peak in the second half of stance as well as for internal lateral knee joint contact forces. The estimation of external joint moments is useful for a general statement about the peak in the first half of stance or for the maximal loading. Nevertheless, when investigating diseases as valgus malalignment, the estimation of lateral knee joint contact forces is necessary for clinical decision making because external joint moments could not predict the lateral knee joint loading sufficient enough. Dependent on the clinical question, either estimating the external joint moments by inverse dynamics or internal joint contact forces by musculoskeletal modeling should be used.

## 1. Introduction

### 1.1. State-of-the-Art

Hip and knee joint osteoarthritis (OA) is a common disease investigated by motion analysis laboratories. Patients with hip or knee OA usually show a different gait pattern (Mündermann et al., 2005; Eitzen et al., 2012), different muscle activities and forces (Loureiro et al., 2013; Rutherford et al., 2013) and also different joint loading (Kaufman et al., 2001; Andriacchi et al., 2004; Foucher, 2017) compared to healthy controls in a similar age. Regarding knee joint OA, an increased external knee adduction moment (KAM) is mainly associated with the progression of medial knee osteoarthritis (mKOA) (Miyazaki et al., 2002; Andriacchi et al., 2004). Patients with hip OA often experience decreased external hip joint moments and especially a decreased hip extension moment (HEM) is significantly correlated with increased pain (Hurwitz et al., 1997). In clinical environments, joint loadings, particularly KAM, are used to conclude about various therapies, e.g., physiotherapy or gait retraining (Shull et al., 2013; van Rossom et al., 2018), treatment with insoles or orthoses (Lindenfeld et al., 1997; Tokunaga et al., 2016), but also for surgeries (Prodromos et al., 1985).

**Figure 1:**
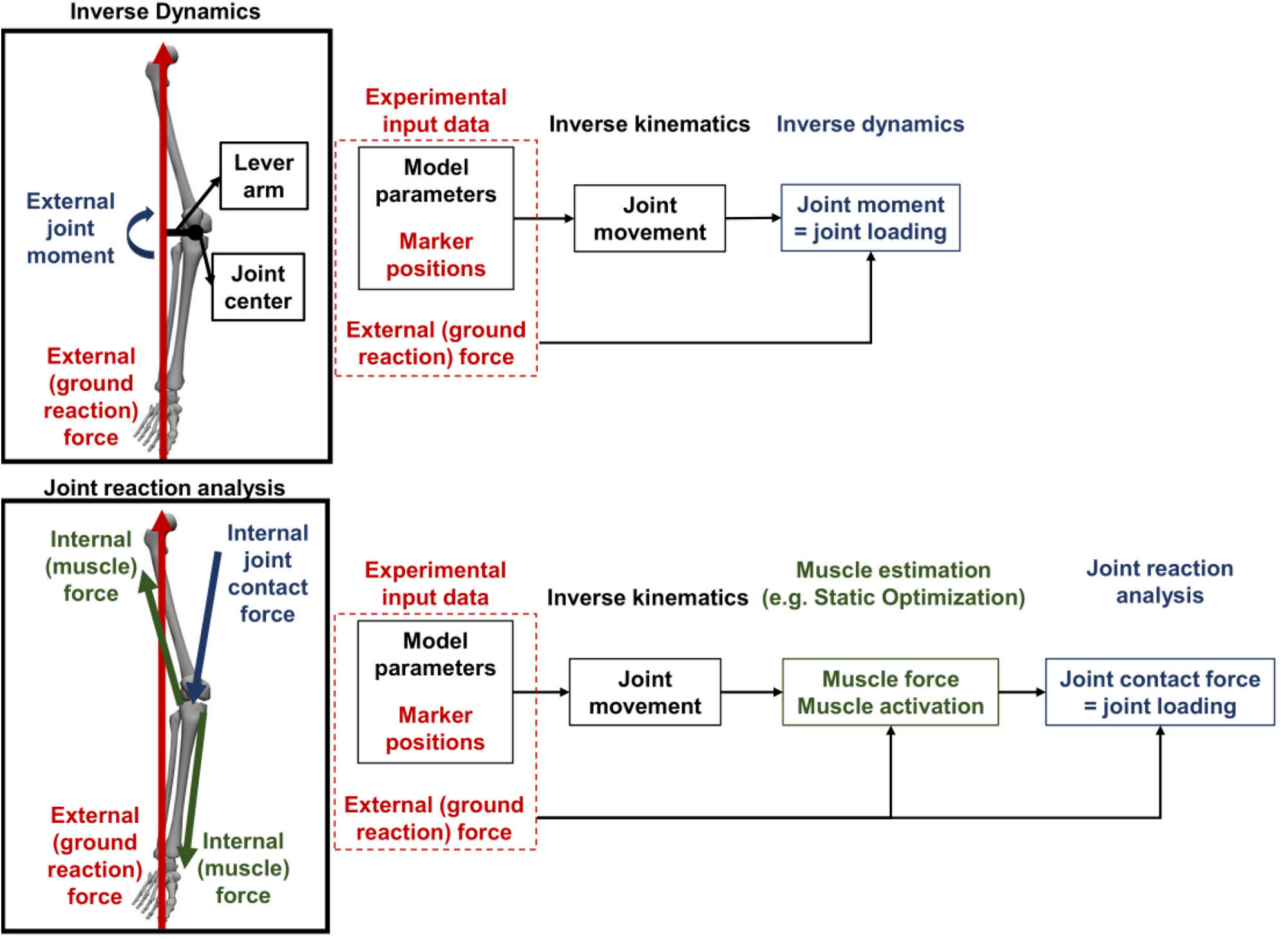
Simplified schematic comparison of inverse dynamics (ID) and musculoskeletal (MSK) modeling for calculating the internal joint contact forces. ID only takes external forces as the ground reaction force (GRF) and a lever arm from the joint center to the GRF vector into consideration when calculating the joint moment as surrogate measure for joint loading. In contrast, with MSK modeling not only the external forces but also internal (muscle) forces are considered for calculating the internal joint contact forces as the representation of the joint loading.

Inverse dynamics (ID) is the mainly used approach to calculate external joint moments allowing the differentiation of moments around the three anatomical axes (frontal, sagittal and transverse axis) of a specific joint center. External joint moments are calculated using external forces (ground reaction forces) which are applied to the body, the kinematics of the joints, the distance from the force vector to the mass center and moments of inertia about the mass center. These parameters are the input variables for the equations of motion which define the underlying model (Pandy and Berme, 1988) (Figure 1). Nevertheless, in patients with valgus malalignment of the lower limb, the loading not only around the knee joint center is important to know, but separately for the medial and lateral compartments. A valgus malalignment increases the internal knee joint contact force (KCF) in the lateral compartment of the knee joint and decreases the KCF in the medial compartment (Holder et al., 2020). While only taking external knee joint moments around a joint center into consideration, these differentiation cannot be made. However, inverse dynamics (ID) is still the most frequently used approach for evaluating the joint loading in clinical gait analysis.

For investigating the above mentioned example of valgus malalignment, other approaches for estimating the joint loading can be considered. Musculoskeletal (MSK) modeling, performed with specific software (e.g., OpenSim or Anybody), is able to do so by estimating the internal joint contact forces. The internal joint contact forces not only take into account the externally applied forces which are also considered by ID, but also the internally applied forces from muscles which act on the joint (Steele et al., 2012) (Figure 1). Even more detailed models also include ligament forces working on the specific joint (Steele et al., 2012). Besides a more detailed calculation, special knee models are available allowing the estimation of medial and lateral compartment loading individually (Winby et al., 2009; Lerner et al., 2015). Moreover, with appropriate medical CT or MRI images, musculoskeletal (MSK) models can be individualized according to the participant (Davico et al., 2020), while experimental electromyography (EMG) data give information about the actual participant-specific muscle activity and muscle force (Pizzolato et al., 2015). This is a clear advantage over ID, because ID does not consider muscle forces or muscle activities (i.e., internal forces) in the calculation.

One problem using MSK modeling for estimating internal joint loads is mainly the higher computational cost in data processing and modeling. In contrast, the calculation of the external joint moments using ID is included in the main processing routine after a classical gait analysis, thus, joint moments are available directly after data collection. MSK modeling software offer generic models based on a single person and anthropocentric information from the literature (Delp et al., 2007). The generic models are scaled to fit the participants body weight (BW) and height using information of a static or dynamic trial. In most of the modeling software, scaling is performed manually which is time consuming and might induce subjectivity. It was shown, that models based on medical images reduce the error of joint moment or joint contact force calculations compared to data from instrumented prosthesis (Wesseling et al., 2016). While medical images are included for scaling, the time for creating a participant-specific model increases (Davico et al., 2020). In marker based scaled models, errors in joint loading or muscle forces (Martelli et al., 2015b) can occur based on soft tissue artifacts (Wesseling et al., 2016) or incorrect defined joint centers (Martelli et al., 2015c; Kainz et al., 2017; Bahl et al., 2019). Moreover, the different definitions of the joints in case of degree of freedom (DoF) or muscle positioning have been shown to influence the outcome (Valente et al., 2014, 2015). A study comparing the outcome of an Anybody and OpenSim model relied the discrepancies mainly on the different muscle definitions and segmental coordinate systems (Trinler et al., 2019). Especially in a clinical environment, time consuming MSK modeling can hardly be performed, because the results need to be available soon after the gait analysis for, e.g., surgery or rehabilitation planning.

Apart from MSK modeling, internal joint contact forces and moments can be directly measured using instrumented prosthesis. Only a few studies exist investigating internal joint contact forces from instrumented prostheses in patients with total hip replacement or total knee replacement (TKR) (Mündermann et al., 2008; Arami et al., 2013; Bergmann et al., 2016). This approach is thought to be the most accurate approach compared to MSK modeling and ID (Schellenberg et al., 2018). Additionally, internal joint contact force distribution can be measured individually with different measuring elements installed in the instrumented prosthesis. Though, patients equipped with an instrumented prosthesis (patients after joint replacement) are rare. Additionally, it is known, that patients after total hip replacement or TKR walk with different gait pattern compared to healthy controls (Meyer et al., 2018; Ro et al., 2020). Therefore, the internal joint contact forces measured in patients after total joint replacement cannot be directly compared to other populations because for these groups data from internal instruments are not available. Nevertheless, forces and moments measured with instrumented prostheses allow the validation of MSK modeling approaches with internally measured data (Schellenberg et al., 2018).

While above calculations all claim to estimate joint loading, it is not clear, however, if a direct general relationship between internal joint contact forces and external joint moments exist. It has not yet been discussed to what extent the calculation of the external joint moments is sufficient to determine the internal joint contact forces.

### 1.2. Research Question and Goals

The goal of this systematic review is, therefore, to examine the quantitative relationship between external joint moments and internal joint contact forces at the hip and knee during walking. Both parameters are used to predict joint loading. However, it is not clear whether the different methodological approaches lead to the same results and clinical conclusions. Furthermore, it is unclear whether it is sufficient to determine external joint moments to draw conclusions regarding internal joint loading. To be able to give a precise overview a clear definition of what we define as external and internal joint loading is necessary, similar to Vigotsky et al. (2019). “External joint moments” or “external joint forces” are defined as parameters which are calculated using the ID approach. “Internal joint moments” and “internal joint contact forces”, on the other hand, define parameters which describe internal loads calculated with MSK modeling or measured with an instrumented prosthesis.

The systematic review shall give a general overview related to the relationship between external joint moments and internal joint contact forces during walking. We additionally aim to identify factors affecting the relationship, e.g., study population or used methods, for which clinical decisions made based on external joint moments would lead to other conclusions compared to the usage of internal joint contact forces. Finally, we targeted to draw conclusions on the preferable method, i.e., ID or MSK modeling, and on corresponding parameters, i.e., external joint moments or internal joint contact forces, for clinical reasoning in gait analysis.

The systematic review is registered at PROSPERO (CRD42020160805) (https://www.crd.york.ac.uk/prospero/display_record.php?RecordID=160805).

## 2. Methods and Materials

### 2.1. Search Strategy and Study Selection

The electronic databases “Pubmed” and “Web of Science” were searched for articles fitting into the inclusion criteria. The search included the key words “(gait OR walk) AND (hip OR knee) AND (force OR moment OR torque) AND (model OR musculoskeletal OR musculosceletal) (inverse dynamics OR simulation)”. The complete search terms can be found in the Supplementary Material (“*A Full search terms”*).

Studies published in English or German as full text versions with abstracts in a peer-reviewed journal have been included in this systematic review. The studies were published between January 1, 1990 (first attempt to model walking with MSK models Pandy and Berme, 1988; Delp et al., 1990) and October 31, 2019. Systematic reviews or meta-analysis, randomized controlled trials, research studies or articles and case reports or series were included. Participants had to be healthy or with orthopedic diseases, e.g., OA in the hip or knee joint. Additionally, participants with an instrumented prosthesis at the hip or knee joint were included, while, in general, participants of all ages were accounted for if they were able to walk independently, without walking aids, e.g., insoles, crutches, braces or an exoskeleton, or any other assistance. Furthermore, data of barefoot walking or walking with defined shoes were analyzed. For data collection, a three-dimensional multi-camera system with integrated force plates able to accurately capture reflective marker data and ground reaction forces had to be used. Joint moments and joint contact forces had to be presented and calculated with an ID approach, with MSK modeling or using an instrumented prosthesis. A quantitative relationship between joint moments and joint contact forces had to be calculated and statistically analyzed.

### 2.2. Quality Assessment

Titles of all qualified articles from the database search were screened by one reviewer (JH). Duplicates and papers without an abstract or full text version available were eliminated. Titles and abstracts were assessed and excluded by two independent reviewers (JH, UT) if not fitting the above mentioned inclusion criteria. All full text versions were checked by two independent reviewers (JH, UT) for inclusion eligibility. Data extraction was performed by one researcher (JH). The data of the articles were only extracted when fulfilling the inclusion criteria. If full text articles were not accessible, authors were contacted. The reviewers were not blinded on title or author names of the studies. One reviewer (JH) screened each study for bias. The quality assessment checklist, adopted from Downs and Black (1998), reviews each included study according to parameters like reporting methods, external and internal validity and was performed by both reviewers (JH, UT).

Data extraction of included studies was documented in one central excel spreadsheet. It contained the following information: author, title, and year of publication, the cohort of the study (healthy or orthopedic patients, children or adults), anthropometric parameters (age, height, weight, body mass index (BMI), knee joint alignment), the measuring equipment (cameras, force plates, when necessary instrumented prosthesis or EMG), data processing, the applied models and software for calculating joint angles, moments and joint contact forces, data of extracted joint moment and joint contact force values (peak(s), total maximum or mean, whole stance or parts of stance, standard deviation), the statistical method and the outcome measures (walking speed, statistical relationship (*r*/*R*^2^ and/or *p*-value, root mean squared error (RMSE)), and existence and content of discussion and conclusion.

The quality assessment checklist covered points related to predefined parameters and categories (see Supplementary Table 1). In total 68 parameters were rated in 9 categories and a total points of 75 was reachable. The categories were: Aims & study population; patients; controls; cameras, markers & force plates; EMG; external loads (angles and moments); internal loads (joint contact forces); general & statistics; discussion & conclusion. A total score (in %) for every category and an overall total score (in %) were calculated for every study dividing the reached points per category with the maximal possible points in this category. The overall score of each paper was calculated by dividing the total reached points with the maximal possible points. A maximal score of 100% could be reached. Both reviewers (JH, UT) rated the included studies independently, while the final score of each paper was the average score of both raters. Large discrepancies in scores between the two reviewers were discussed and in case of disagreement a third reviewer was planned to be consulted, which, however, was not necessary. It must be mentioned that this quality assessment scoring concentrated on the performed and reported methods to estimate the external and internal joint loading parameters and to estimate the statistical relationship between these measures. The scoring does not rank how well the studies were performed. Therefore, as recommended by the guidelines of Cochrane, we do not categorize the studies according to the total scores (https://handbook-5-1.cochrane.org/chapter_8/8_3_2_reporting_versus_conduct.htm).

### 2.3. Calculation of Joint Loading Parameters

As already stated in the previous subsection, joint moments and joint contact forces had to be calculated either by an ID approach, by MSK modeling or with an instrumented prosthesis. Extracted joint loading parameters from ID were classified as external joint loading measures. Parameters from MSK modeling which also take internal forces, e.g., muscle forces, into consideration when calculating the internal joint contact forces, or joint loading parameters directly measured with the instrumented prosthesis, were categorized as internal joint loading parameters (see Figure 1). To be able to compare the different studies, the detailed description of the used models were extracted. We focused on the model description, segments and joint DoF, the applied software and the used methods to calculate the external and internal joint loading parameters. Additionally, the values which were used for the statistical analysis were extracted (e.g., peak values or the mean of a parameter).

### 2.4. Statistical Analyses of the Relationship Between External and Internal Joint Loading Measures

Studies were only included if a statistical relationship between joint moments and joint contact forces was calculated and statistically analyzed. Therefore, either *r*-, *R*^2^-values and/or the *p*-value, the RMSE or other statistical output parameters had to be available. To compare the output between studies, a separate spreadsheet was used in which only the statistical output of included studies were summarized. The findings were divided into several parts: Results for peak values (e.g., medial knee joint contact force (mKCF)) in the first half of stance or in the second half of stance as well as results for overall peak values. *R*^2^-values below 0.25 were interpreted as low, between 0.25 and 0.49 as moderate and above 0.49 as high similar to Hinkle et al. (1988) and Kotrlik and Williams (2003).

## 3. Results

### 3.1. Search Strategy Yield and Quality Assessment

**Figure 2:**
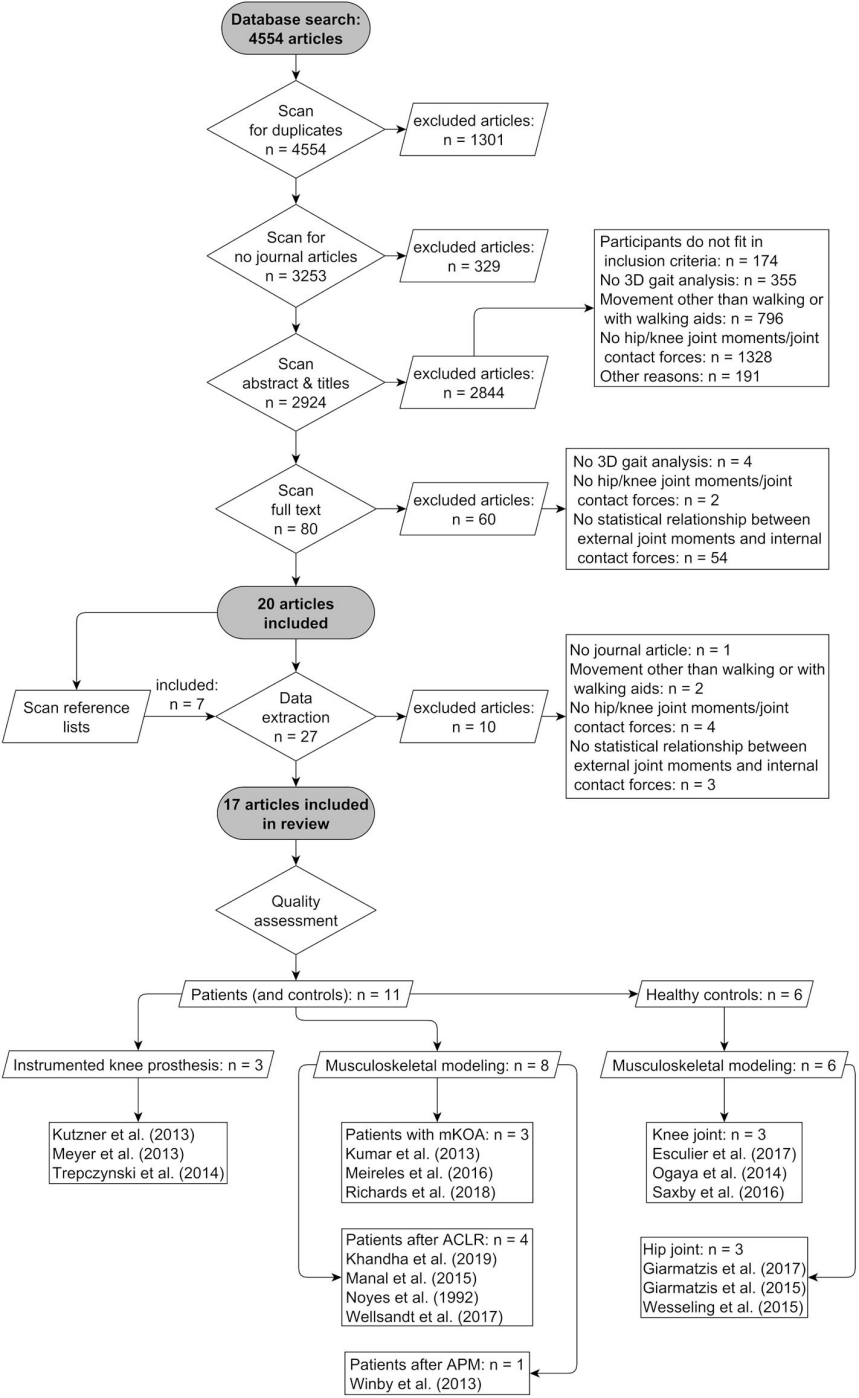
Flow diagram of study selection and results. *mKOA*, medial knee osteoarthritis; *ACLR*, anterior cruciate ligament reconstruction; *APM*, arthroscopic partial meniscectomy.

Figure 2 presents the study selection process and the final outcome. Searching the databases yielded a total of 4,554 studies. After scanning for duplicates and publication types other than journal articles, a total of 3,253 studies were included in the title, abstract and full text scanning. In the end, 17 studies were included in the systematic review for quality assessment. A total of 14 studies evaluated the relationship between external and internal joint loading parameters at the knee joint and three studies at the hip joint. Of the 14 studies that investigated the relationship at the knee joint, three studies assessed patients according to TKR with instrumented knee prosthesis (Kutzner et al., 2013; Meyer et al., 2013; Trepczynski et al., 2014). Seven additional studies investigated either patients with mKOA (Kumar et al., 2013; Meireles et al., 2016; Richards et al., 2018) or after anterior cruciate ligament reconstruction (ACLR) (Noyes et al., 1992; Manal et al., 2015; Wellsandt et al., 2017; Khandha et al., 2019). In one study, patients were evaluated approximately 16 weeks after arthroscopic partial meniscectomy (APM) (Winby et al., 2013). Three studies analyzed the relationship at the knee joint in healthy participants (Ogaya et al., 2014; Saxby et al., 2016; Esculier et al., 2017). The remaining three studies studied the hip joint relationship (Giarmatzis et al., 2015, 2017; Wesseling et al., 2015). The detailed description of the anthropometric data of the study populations and the methodology used to determine the internal joint contact forces are summarized in Table 1.

**Table 1:** Summary of the study population characteristics and methods of calculating the internal joint contact forces.

**Study**	**Population**	**No. (m/f)**	**Age [years]**	**BMI [kg/m^2^]**	**Internal joint contact forces**
**Knee joint**					
Kutzner et al., 2013	Patients after TKR	9 (6/3)	69.9 ± 4.8	30.6 ± 4.3	Instrumented prosthesis
Meyer et al., 2013	Patients after TKR	1 (1/0)	83.0	24.2	Instrumented prosthesis
Trepczynski et al., 2014	Patients after TKR	9 (6/3)	70.0 ± 5.0	30.6	Instrumented prosthesis
	Patients with mKOA	16 (8/8)	65.2 ± 9.5	28.6 ± 4.3	EMG-informed MSK model
Kumar et al., 2013	Controls	12 (6/6)	59.5 ± 10.4	28.4 ± 5.2	EMG-informed MSK model
Meireles et al., 2016	Patients with early mKOA	16 (0/16)	64.9 ± 6.0	-	MSK modeling
	Patients with established mKOA	23 (0/23)	65.6 ± 7.2	-	
	Controls	20 (0/20)	64.6 ± 8.7	-	
Richards et al., 2018	Patients with mKOA	35 (13/22)	62.3 ± 5.9	25.5 ± 2.6	MSK modeling
	Patients after ACLR	36 (23/13)	29.0 ± 10.0	21.1	
Khandha et al., 2019	Controls	12 (7/5)	23.0 ± 5.0	26.0	EMG-informed MSK model
Manal et al., 2015	Patients after ACLR	10 (5/5)	30.1 ± 7.9	28.8	EMG-informed MSK model
	Patients after ACLR	32 (20/12)	27.0 (15-41)	-	
Noyes et al., 1992	Controls	16 (9/7)	26.0 (19-45)	-	Mathematical model
Wellsandt et al., 2017	Patients after ACLR	30 (19/11)	30.5 ± 11.1	26.7 ± 4.0	EMG-informed MSK model
Winby et al., 2013	Patients after APM and controls	27	46 ± 6	25.3	EMG-informed MSK model
Esculier et al., 2017	Controls	87 (51/36)	23.0 ± 3.8	23.0 ± 3.1	Mathematical model
Ogaya et al., 2014	Controls	122 (31/91)	73.8 ± 6.3	21.6	MSK modeling
Saxby et al., 2016	Controls	60 (35/25)	27.3 ± 5.4	22.8	EMG-informed MSK model
**Hip joint**					
	Controls (young)	14 (0/14)	21.4	22.6	
Giarmatzis et al., 2017	Controls (elderly)	14 (0/14)	69.6	24.4	MSK modeling
Giarmatzis et al., 2015	Controls	20 (10/10)	22.2 ± 1.6	21.5 ± 1.7	MSK modeling
Wesseling et al., 2015	Controls	5 (2/3)	56.0 (52-61)	22.3 ± 1.6	MSK modeling

*BMI, body mass index; MSK, musculoskeletal; EMG, electromyography; ACLR, anterior cruciate ligament reconstruction; mKOA, medial knee osteoarthritis; TKR, total knee replacement; APM, arthroscopic partial meniscectomy*.

Table 2 reveals the scores of the quality assessment. The total score was 80 ± 10% and varied between 61 and 93%. The largest variance between scores was found for the categories “Equipment” (75 ± 24%), “External joint moments/forces” (77 ± 20%) and “Internal joint contact forces” (74 ± 25%).

**Table 2:** Results of the quality assessment screening for each study and additionally a total score is showed in %.

**Study**	**Aims**	**Pat**.	**Cont**.	**Equip-** **ment**	**EMG**	**Ext**.	**Int**.	**Stat**.	**Disc. &** **conc**.	**Tot**.
**Knee joint**										
Kutzner et al., 2013	50	90	-	54	-	83	54	89	100	74
Meyer et al., 2013	50	65	-	19	75	70	39	67	100	61
Trepczynski et al., 2014	50	90	-	54	-	50	43	61	100	64
Kumar et al., 2013	100	100	91	46	88	55	55	67	100	78
Meireles et al., 2016	100	83	82	62	-	100	100	100	100	91
Richards et al., 2018	75	88	-	92	-	100	100	67	100	89
Khandha et al., 2019	75	92	-	92	100	55	55	94	100	83
Manal et al., 2015	75	92	-	87	100	85	90	100	100	91
Noyes et al., 1992	100	67	55	46	-	50	30	83	100	66
Wellsandt et al., 2017	50	75	-	81	100	70	80	89	100	81
Winby et al., 2013	88	56	55	92	100	68	85	56	100	78
Esculier et al., 2017	75	-	82	92	-	40	43	94	100	75
Ogaya et al., 2014	50	-	91	92	-	90	90	89	100	86
Saxby et al., 2016	75	-	91	100	100	100	90	89	100	93
**Hip joint**										
Giarmatzis et al., 2017	88	-	55	100	-	98	100	78	100	88
Giarmatzis et al., 2015	50	-	91	100	-	100	100	94	100	91
Wesseling et al., 2015	50	-	59	65	-	90	100	78	100	77
**Mean** **±** **SD**	71 ± 19	81 ± 13	75 ± 16	75 ± 24	95 ± 9	77 ± 20	74 ± 25	82 ± 14	100 ± 0	80 ± 10

*Pat., patients; Cont., controls; Ext., external joint moments/forces; Int., internal joint contact forces; Stat., General information and statistics; Disc. & conc., discussion and conclusion; Tot., total score; SD, standard deviation*.

### 3.2. Statistical Methods

**Figure 3:**
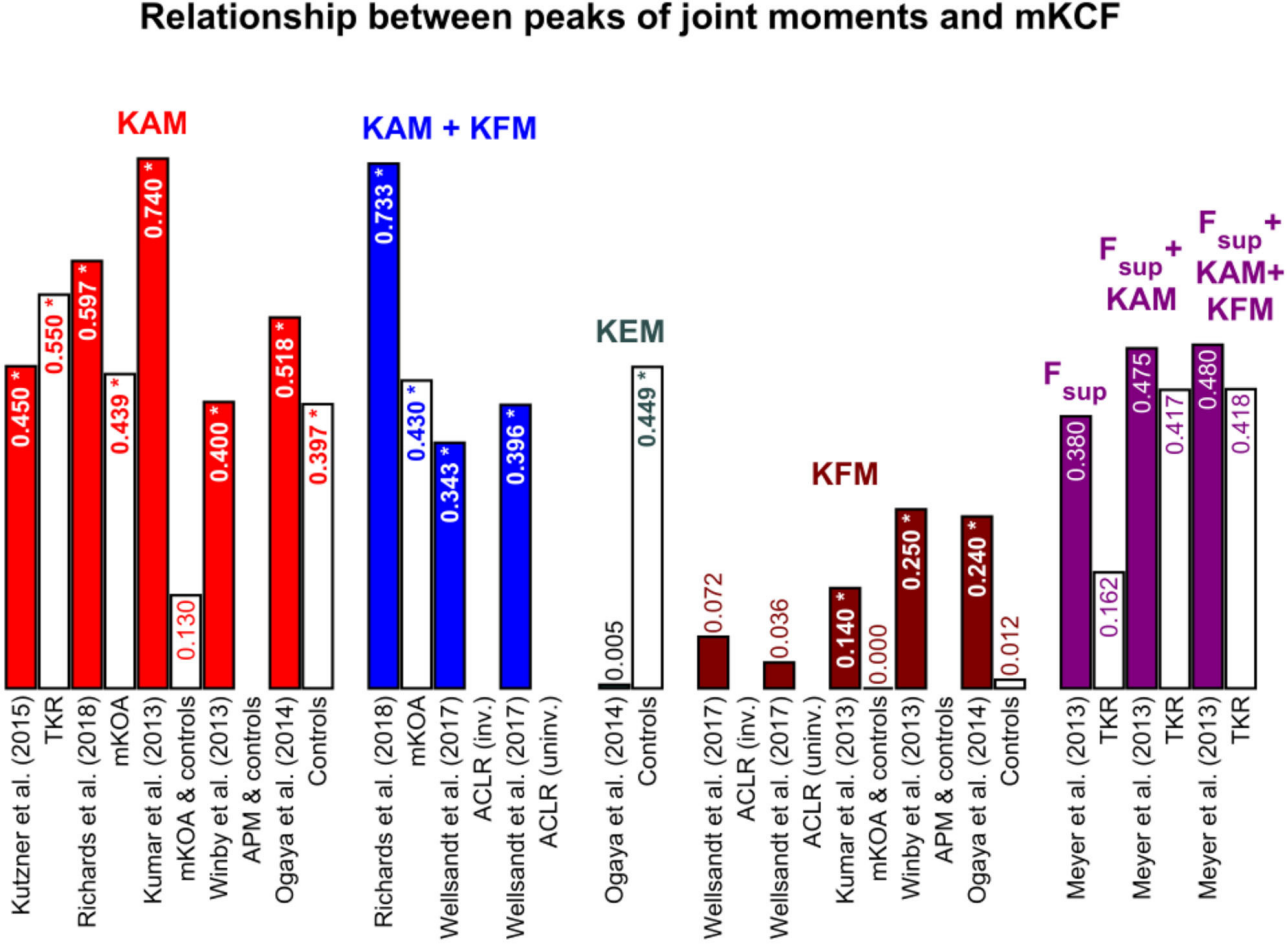
Coefficient of determinations between the first and second peaks of the joint moments and medial knee joint contact force (mKCF). The * indicates significant correlations. The exact *p*-values can be found in the Supplementary Table 4. Colored bars represent the findings for the first peak and the white bars with colored text for the second peak of the same study. *KAM*, knee adduction moment; *KFM*, knee flexion moment; *KEM*, knee extension moment; *F_sup*, superior force; *TKR*, Patients after total knee replacement; *mKOA*, Patients with medial knee osteoarthritis; *APM*, Patients after arthroscopic partial meniscectomy; *ACLR*, Patients after anterior cruciate ligament reconstruction; *inv*., involved leg; *uninv*., uninvolved leg.

**Figure 4:**
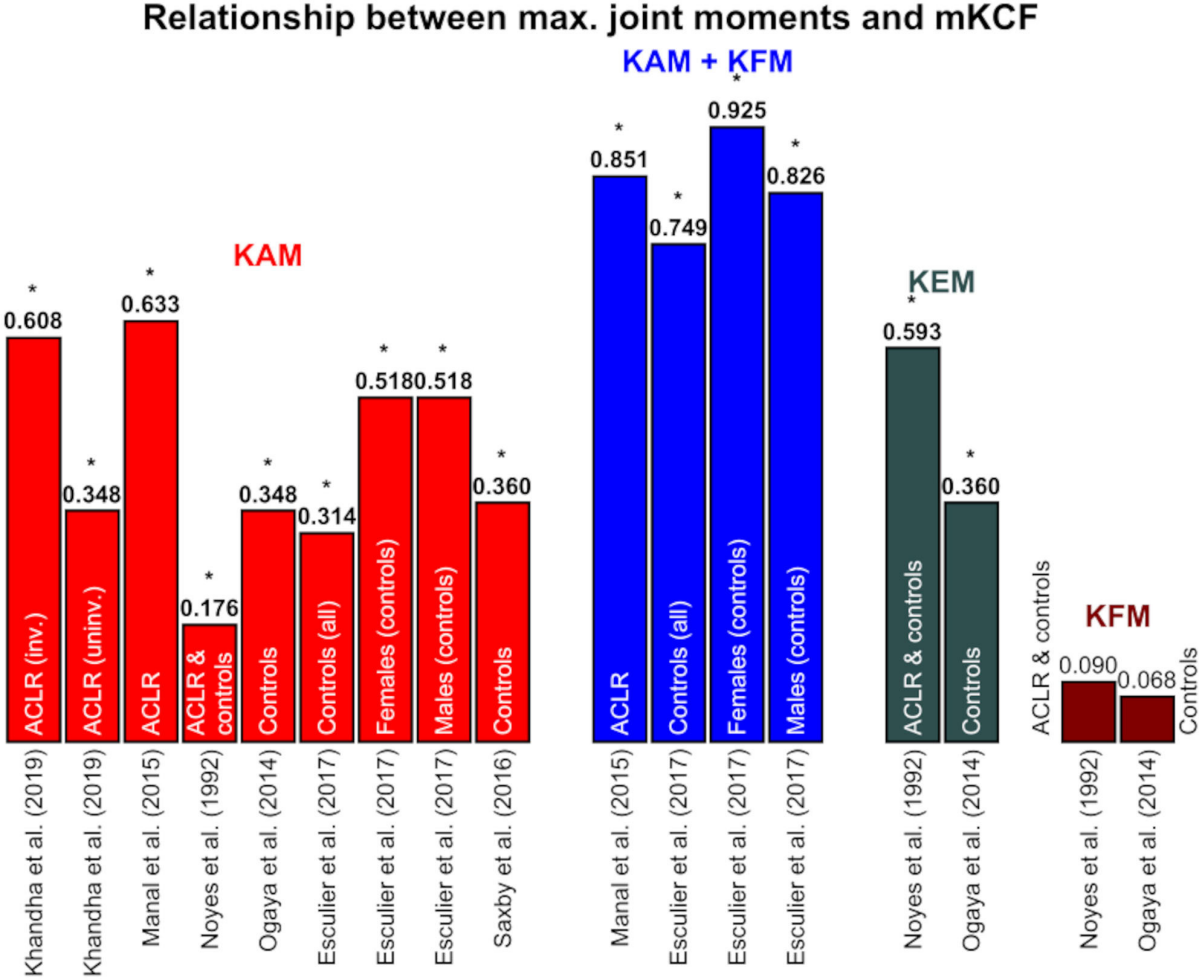
Coefficient of determinations between the maximal values of the joint moments and medial knee joint contact force (mKCF). The * indicates significant correlations. The exact *p*-values can be found in the Supplementary Table 5. *KAM*, knee adduction moment; *KFM*, knee flexion moment; *KEM*, knee extension moment; *ACLR*, Patients after anterior cruciate ligament reconstruction; *inv*., involved leg; *uninv*., uninvolved leg.

**Figure 5:**
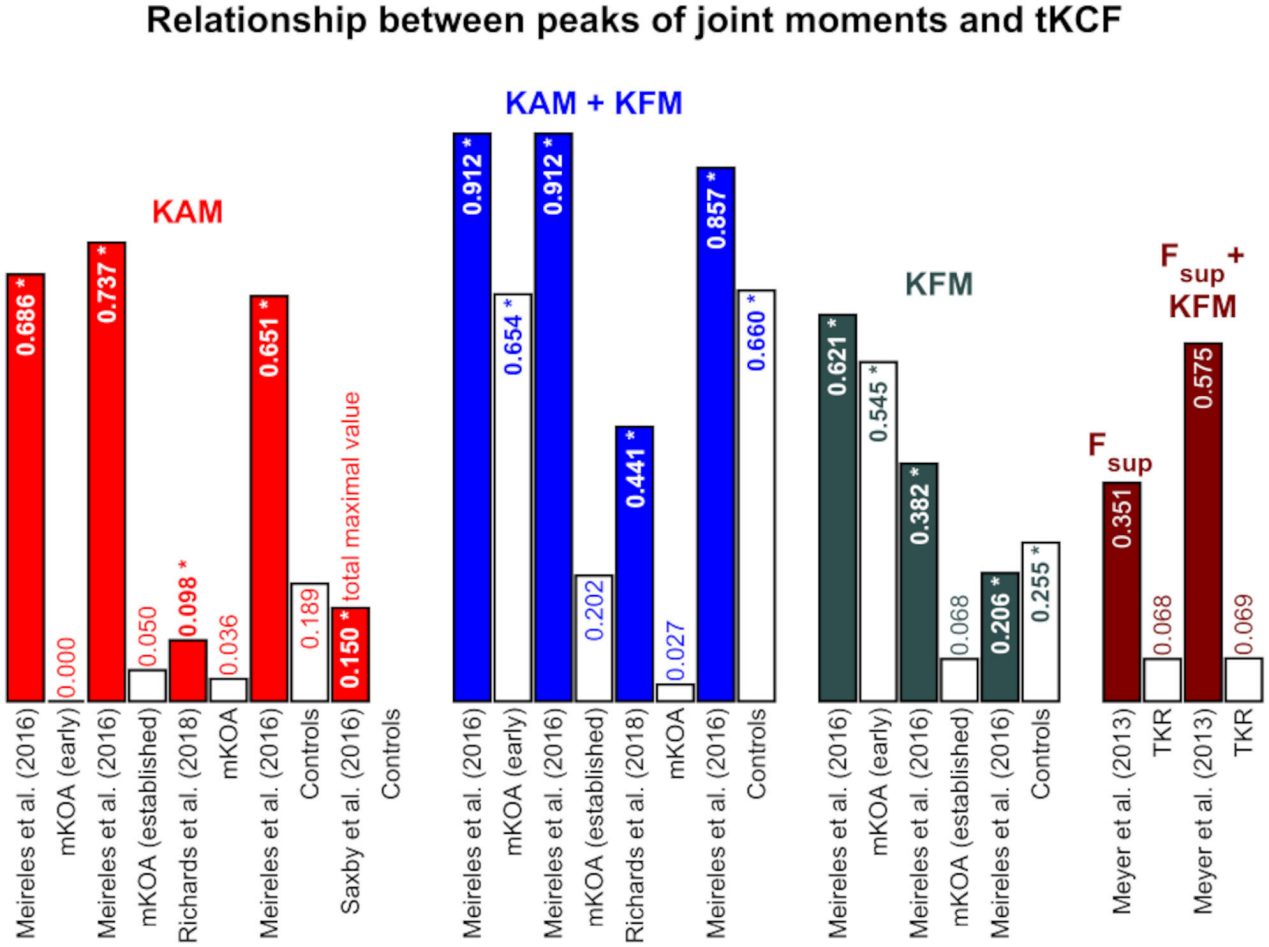
Coefficient of determinations between the first and second peaks of the joint moments and total knee joint contact force (tKCF). The * indicates significant correlations. The exact *p*-values can be found in the Supplementary Table 6. Colored bars represent the findings for the first peak and the white bars with colored text for the second peak of the same study. *KAM*, knee adduction moment; *KFM*, knee flexion moment; *F_sup*, superior force; *TKR*, Patients after total knee replacement; *mKOA*, Patients with medial knee osteoarthritis.

The applied statistical analyses were well-reported in most of the studies with an average score of 84%. All studies conducted a correlation or linear regression analysis with various input (external and internal joint loading parameters) and output variables (*r*-/*R*^2^ and *p*-values or other parameters). Only Trepczynski et al. (2014) did not set *r*-/*R*^2^ or *p*-values individually for walking. They also performed movements other than walking, such as ascending or descending stairs, and calculated a *R*^2^ and *p*-value for all activities together. Therefore, we included this study in the systematic review, but not in the summary tables for the reported relationships. Kumar et al. (2013) performed a multiple regression analysis and reported the *p*-value and the β value, a standardized regression coefficient that can be compared to *r*-values when only one independent variable is used in the multiple regression analysis. For better understanding, the *R*^2^ values were calculated from the original *r*-values if the *R*^2^ values were not specified by the authors. The calculated *R*^2^ values are highlighted in blue. Most authors did not report the relative time of the gait cycle at which they extracted the total maximal values. Therefore, the results for the first or second peak and the total maximal value were reported separately in different figures and tables (Figures 3–5, and Table 3). A summary table of the performed statistical analyses and the extracted statistical parameters is included in the Supplementary Table 2).

**Table 3:** Results for lateral knee contact force: Relationship for the total maximum, first, and second peaks.

**Study**	**Population**	**Independent variable**	**Total maximal value**		**First peak**		**Second peak**
			***R^2^***	***p***	***R^2^***	***p***	***R^2^***
Noyes et al., 1992	Patients (ACLR) & controls	KAM	0.002	> 0.05			
Winby et al., 2013	Patients (APM) & controls	KAM			0.12	<0.05	
Noyes et al., 1992	Patients (ACLR) & controls	KFM	0.096	> 0.05			
Winby et al., 2013	Patients (APM) & controls	KFM			0.29	<0.05	
Noyes et al., 1992	Patients (ACLR) & controls	KEM	0.810	<0.01			
Saxby et al., 2016	Controls	KAM	0.01	> 0.05			
Meyer et al., 2013	Patients (TKR)	F_sup			0.175		0.002
		F_sup + KAM			0.797		0.006
		F_sup + KAM + KFM			0.822		0.007

*ACLR, anterior cruciate ligament reconstruction; APM, arthroscopic partial meniscectomy; KFM, knee flexion moment; KEM, knee extension moment; KAM, knee adduction moment; F_sup, superior force*.

*Values in blue: R^2^ is calculated from original r-value*.

### 3.3. Estimation of External Joint Moments and Internal Joint Contact Forces

All included studies used ID to determine the external joint moments and forces. Different approaches, however, were used to calculate the internal joint contact forces. In three studies, patients with an instrumented prosthesis were examined to directly measure the internal joint contact forces. In two studies, the same type of instrumented prosthesis consisting of 6 strain gauges was used (Kutzner et al., 2013; Trepczynski et al., 2014). As a result, 3 force and 3 moment components were analyzed, of which the axial force was transmitted through the medial and lateral compartments. Meyer et al. (2013) used an implant also consisting of 6 DoFs (3 for the force and 3 for the moment components) but the geometry varied slightly compared to the instrumented prosthesis used in the other 2 studies.

The studies, which used an EMG-informed MSK model (Kumar et al., 2013; Winby et al., 2013; Manal et al., 2015; Saxby et al., 2016; Wellsandt et al., 2017; Khandha et al., 2019), based their calculations of the internal KCF on the same equations (Lloyd and Besier, 2003; Winby et al., 2009), which allow separate calculation of the medial and lateral compartmental loading. An extension (Lloyd and Buchanan, 1996) of the generic 1 DoF knee model (Delp et al., 1990) was used as the anatomical model. This EMG-informed model contains in the included studies participant-specific EMG data of the medial and lateral hamstrings, medial and lateral vastii, medial and lateral gastrocnemii and the rectus femoris (Kumar et al., 2013; Manal et al., 2015; Wellsandt et al., 2017; Khandha et al., 2019), additionally of the tensor fascia latae (Saxby et al., 2016), the sartorius and the gracilis (Winby et al., 2013). Two other studies (Ogaya et al., 2014; Meireles et al., 2016) based their calculations on the same generic OpenSim model “gait2392” with the 1 DoF generic knee model (Delp et al., 1990, 2007). Richards et al. (2018) were the only investigators using the Anybody software and the corresponding Twente Lower Extremity Model (TLEM) with a 1 DoF knee model (Klein Horsman et al., 2007; Carbone et al., 2015). In this model, ligament and muscle forces were included in the calculation of the knee joint loading. Noyes et al. (1992) was the oldest study presented in this systematic review and was based on the calculations according to Schipplein and Andriacchi (1991) which allowed rotation about an axis (flexion-extension) and the calculation of mKCF as a proportion of total knee joint contact force (tKCF). As in the previous model, ligament forces from the medial and lateral collateral ligament were also included in the calculation of the internal KCF. Esculier et al. (2017) used a different knee model considering quadriceps, hamstrings and gastrocnemius muscle forces (DeVita and Hortobagyi, 2001; Messier et al., 2011). The proportion of mKCF was estimated using the equations from Schipplein and Andriacchi (1991). All studies that analyzed the relationship between external and internal joint loading measures at the hip joint (Giarmatzis et al., 2015, 2017; Wesseling et al., 2015) applied either the generic “gait2392” OpenSim model (Delp et al., 1990, 2007) or another OpenSim model for the lower extremities (Hamner et al., 2010) that was also based on the generic “gait2392” model. The hip joints in these models were modeled as 3 DoF ball joints.

### 3.4. Relationship Between External and Internal Joint Loading

The studies mainly examine the internal mKCF (Figures 3, 4). Four studies additionally studied lateral knee joint contact force (*l*KCF) (Table 3), while four other studies explored the relationship between external knee joint loading parameters and tKCF (Figure 5). The studies investigated the relationship between internal KCF and external KAM and/or the external knee flexion moment (KFM)/knee extension moment (KEM). The transverse plane was not considered. Meyer et al. (2013) looked into the relationship between a superior force (F_sup) acting on the knee joint in combination with external knee joint moments (KEM/KFM) and the internal tKCF. Here, F_sup described the external force applied by the ground reaction force along the vertical axis of the shank.

Mainly, the relationship at the peak in the first and/or second half of stance (Figures 3, 5 and Table 4) were considered, while a few studies examined the relationship between the total maximal values (Figure 4 and Table 3). Not all of the latter studies provided information about the time at which the total maximal value occurred, which is why we analyzed the total maximal value independently of the values of the first and second peaks—although they may occur at similar times in the gait cycle than the first or second peak.

**Table 4:** Summary table of the findings about the relationship between external knee joint moments and internal mKCFs and tKCFs.

			**KAM**	**KFM**	**KEM**	**KAM + KFM**
		First peak	Moderate	-	-	-
	mKCF	Second peak	Strong	-	-	-
Patients after TKR		Maximal	-	-	-	-
		First peak	-	-	-	-
	tKCF	Second peak	-	-	-	-
		Maximal	-	-	-	-
		First peak	Strong	-	-	Strong
	mKCF	Second peak	Moderate	-	-	Moderate
Patients with mKOA		Maximal	-	-	-	-
		First peak	Strong	Strong	-	Strong
	tKCF	Second peak	Low	Moderate	-	Moderate
		maximal	-	-	-	-
		first peak	-	x	-	Moderate
	mKCF	Second peak	-	-	-	-
Patients after ACLR		Maximal	Strong	-	-	Strong
		First peak	-	-	-	-
	tKCF	Second peak	-	-	-	-
		Maximal	-	-	-	-
Patients after APM	mKCF	First peak	Moderate	Low	-	-
		First peak	Strong	Low	x	-
	mKCF	Second peak	Strong	x	Moderate	-
Healthy controls		Maximal	Moderate	Low	Moderate	Strong
		First peak	Strong	Low	-	Strong
	tKCF	Second peak	x	Moderate	-	Strong
		Maximal	Low	-	-	-

*The predictability of the internal joint contact forces is classified in four different stages: -: relationship was not investigated; x: no predictability (in red); low: low predictability (in orange); moderate: moderate predictability (in yellow); strong: strong predictability (in green). The stages are related to the investigated R^2^-values which were interpreted as low below 0.25, between 0.25 and 0.49 as moderate and above 0.49 as high Hinkle et al. (1988); Kotrlik and Williams (2003)*.

*KAM, knee adduction moment; KFM, knee flexion moment; KEM, knee extension moment; mKCF, medial knee joint contact force; tKCF, total knee joint contact force; TKR, total knee replacement; mKOA, medial knee osteoarthritis; ACLR, anterior cruciate ligament reconstruction; APM, arthroscopic partial meniscectomy*.

#### 3.4.1. Medial Knee Joint Contact Force

Moderate to strong correlations between mKCF and KAM were observed for the peak in the first half of stance across all populations included. For the peak in the second half of stance, however, a less strong relationship was mainly detected. Also, only low associations were noted between KFM and mKCF for both peaks. The relationship was enhanced when KAM and KFM were combined to predict mKCF.

Significant moderate to strong associations between total maximal values of KAM and mKCF were reported for patients after ACLR as well as for healthy controls. Moderate correlations were detected between the total maximal values of KEM and mKCF, but not between the total maximal values of KFM and mKCF. Again, the relationship was stronger when KAM and KFM were combined to predict mKCF (Figures 3, 4).

#### 3.4.2. Lateral Knee Joint Contact Force

The correlation between *l*KCF and external joint loading measures was only less researched (total: 4 studies). These studies only revealed a low association between KAM or KFM and *l*KCF for the peak in the first half of stance and the total maximal values. Almost no connection was observed for the peak in the second half of stance. A strong relationship was found only between the total maximal KEM and *l*KCF and when combining several external joint loading measures to predict *l*KCF for the peak in the first half of stance (Table 3).

#### 3.4.3. Total Knee Joint Contact Force

Studies examining patients with mKOA and healthy controls mostly reported moderate to strong associations between tKCF and KAM or KFM for the peak in the first half of stance and a stronger correlation for the combination of KAM and KFM. For the peak in the second half of stance, low correlations between KAM and tKCF and stronger interactions between KFM and tKCF were observed (Figure 5).

#### 3.4.4. Hip Joint Contact Force

The external hip adduction moment (HAM) correlated strongly with the internal hip joint contact force (HCF) for all investigated study groups (Giarmatzis et al., 2015, 2017; Wesseling et al., 2015) and for all walking speeds in the first half of stance. Whereas between HCF and HEM and hip rotation moment (HRM), respectively, were predominantly low correlations observed. In contrast, the peak in the second half of stance of HAM showed only a low correlation with HCF, while strong relations were observed for hip flexion moment (HFM) or HEM and partly for HRM. Similar to the results for the knee joint, the relationship can be improved by combining more than one external joint moment to predict HCF (Supplementary Table 3).

## 4. Discussion

The aim of this systematic review was to analyze the relationship between external joint moments and internal joint contact forces at the hip and knee for healthy participants as well as patients (patients after TKR, patients with mKOA, after ACLR or after APM). In total, 14 and 3 studies were found that investigated the relationship between external and internal measures at the knee and hip joint, respectively. External joint moments were calculated using ID while internal joint contact forces were estimated by MSK modeling, other mathematical approaches or measured with an instrumented prosthesis. A meta-analysis was not performed due to the variability of the studied populations and the different clinical questions. Only a few studies were included, so that only a limited number of data sets were available that could be used for a meta-analysis. Furthermore, the aim of this systematic review was to provide a general overview, so we decided to prepare a statistical summary of the results from the included studies.

### 4.1. Relationship Between External and Internal Joint Loading

A schematic summary of the observed correlations between mKCFs or tKCFs and the external knee joint moments are shown in Table 4. In general, for the maximal values and the peak in the first half of stance, mKCFs was best predictable by KAM and in combination with KFM. The *l*KCF was only less strong predicted by knee joint moments throughout the whole stance phase. KAM strongly predicted the tKCFs in the first half of stance and even more accurate together with KFM. Included studies examining the hip joint found similar results compared to the knee joint. The HAM correlated better with the HCF peak in the first half of stance compared to the peak in the second half of stance. When combining several external measures (e.g., HAM and HEM) the relationship was stronger than only with one external measure.

Joint moments and joint contact forces can be divided in its three plane components, meaning that one plane component might not be sufficient enough to explain the full load of a joint. Only one external component might be accurate enough if the joint loading is mostly distributed in one direction. Therefore, describing the internal joint contact forces with more than one external joint moment component (i.e., KAM and KFM) result in a better correlation. Still, the reason that the relationship between KAM and the internal KCFs is frequently investigated, might be because KAM is mainly associated as a surrogate measure for internal KCFs (Andriacchi et al., 2004). An increased KAM during stance was reported as an indicator for increased medial compartment loading and for the progression of mKOA (Miyazaki et al., 2002; Andriacchi et al., 2004). A separate medial and lateral calculation of internal KCF is only possible with MSK modeling or instrumented prosthesis. This might explain why the relationship between external joint moments and internal *l*KCFs were only investigated in 4 previous studies. As a conclusion, we do not recommend to use external joint moments to predict the internal loading in the lateral compartment of the knee joint.

### 4.2. Factors that May Influence the Relationship

#### 4.2.1. Time of Stance

The main difficulty in summarizing the statistical results of included papers was the variety of the point in time of stance which was used to analyze the relationship between external and internal forces. In other words, some studies extracted peak values for the first and/or second peak of stance, others extracted the overall maximal value. Especially, using the total maximal value of the joint moment or joint contact force without reporting the point of time of this value, makes it more difficult to set the findings and conclusions in perspective to the outcome at the first and second peak. Furthermore, only a few studies additionally reported the point in time (% of stance) at which the maximum occurred. Therefore, it was decided to separately report the external-internal force relationship of the first and second peak in stance as well as the overall maximum during stance. For the future we suggest to report the point of time of the extracted values or when possible to use other statistical methods to analyze the whole stance or gait phase and not only one discrete value.

In various findings, an increased first peak knee or hip joint moment was associated with pathological changes (e.g., knee or hip joint OA, Baliunas et al., 2002; Liao et al., 2019) and disease severity (Sharma et al., 1998) or pain (Thorp et al., 2007). This might be the reason, why the first peak was also more often considered in terms of clinical decision making. For these populations, the first peak KAM or HAM can be used as surrogate measure for mKCF respectively HCF. In contrast, the second half of stance phase should be investigated by estimating the internal KCFs while the studies showed less contribution of the hip and knee joint moments in the joint contact forces and therefore a less accurate interpretation of internal joint loading. Additionally, loading in the lateral compartment of the knee joint should be analyzed by using the *l*KCFs throughout the whole stance phase.

For the hip and knee joint, a stronger correlation between internal and external forces was observed for the first peak compared to the second peak. An explanation for this could be the different muscle activities during the gait cycle. The first peak mostly occurs approximately at around 12% of the gait cycle. At this point mainly the vastii muscles (for the knee joint) and gluteus medius and maximus (for the hip joint) are active and contribute mainly to the internal joint contact forces (Pandy and Andriacchi, 2010; Sasaki and Neptune, 2010). Additionally, at this point, the double leg support is still ongoing or the contralateral leg just left the ground, therefore, the stability of the leg should be almost at optimum (Perry and Burnfield, 2010). The second peak usually occurs at the end of terminal stance (at approximately 45% of the gait cycle), where the leading leg is still in single support. At this point, the gastrocnemii muscles move the body forward and, therefore, contribute the most to the joint contact forces (Pandy and Andriacchi, 2010; Sasaki and Neptune, 2010). Moreover, it is assumed that co-activation has a greater influence in the calculation of the internal joint contact forces in the second peak compared to the first peak (Pedersen et al., 1987). This might explain the lower correlation between external joint moments and internal joint contact forces at the second peak. In other words, we can conclude that during first peak the hip and knee spanning muscles are active, giving stability to the joints, which might lead to a better internal-external force relationship.

#### 4.2.2. Differences in Study Population

Five different populations have been, in total, analyzed in included studies (three studies: patients after TKR, three studies: patients with mKOA, four studies: patients after ACLR, one study: patients after APM, 10 studies: healthy controls). In general, more extracted values were compared and analyzed for healthy populations than for patient groups.

Depending on the patient groups, variations in kinematics, kinetics and muscle activation can occur. Patients 24 and 33 months after TKR still exhibit a changed gait pattern and muscle activity compared to age-matched healthy controls (Lundberg et al., 2016; Ro et al., 2020). To add on, patients with mKOA adapted compensatory mechanisms as an increased trunk lean in the direction of the affected limb or a more outward rotated foot compared to healthy controls to reduce the load in the affected joint (Arnold et al., 2014; Kuwahara et al., 2020). Furthermore, patients 2 years after ACLR still performed a different gait pattern and knee joint loading compared to healthy controls (Noehren et al., 2013; Erhart-Hledik et al., 2018). Also, patients 2 years after total hip replacement still show decreased KAMs and increased HAMs for the peak in the second half of stance compared to controls for both the affected and unaffected limb (Stief et al., 2018). Also, gait adaptations altering the external KAM do not necessarily affect internal KCF (Walter et al., 2010; Kinney et al., 2013; Richards et al., 2018). The effect of gait adaptations on internal HCFs have also been investigated. Decreased HCFs were associated with a reduced hip adduction angle which also reduced HAM (Wesseling et al., 2015). Pelvis rotation also highly contributes in HAM (Ardestani et al., 2015). Nevertheless, the direct influence of kinematic changes on the relationship between joint moments and joint contact forces was not yet evaluated. As a result, we recommend examining the effect of gait adaptations separately on joint moments and joint contact forces and, additionally, its direct impact on the relationship between external and internal joint loading measures. Thus, more studies should be performed on different patients investigating the relationship between external and internal joint loading parameters while also considering larger sample sizes.

Previous studies showed an increased KAM (Hurwitz et al., 2002) and mKCF (Smith et al., 2016) in patients with varus malalignment, while a valgus malalignment decreased KAM and increased *l*KCF (Holder et al., 2020). Additionally, the static limb alignment contribute largely on the joint loading distribution on the medial and lateral compartment (Smith et al., 2016). The lower limb alignment was, however, not reported for all participants in the included studies. Kutzner et al. (2013) performed correlation analyses between external knee joint moments and internal KCFs for varus and valgus aligned knee joints separately and did not find significant differences between them. Consequently, we suggest to evaluate the impact of lower limb alignment on the relationship between joint moments and joint contact forces although its effect separately on these parameters was already investigated.

#### 4.2.3. Limitations of Musculoskeletal Modeling

In a few studies, basic information about the used model were missing, e.g., about the number of segments in the model, degrees of freedom or the used software. A previous study showed that diverse coordinate systems were a key factor in contrasting kinematics, kinetics and also muscle activation and forces (Roelker et al., 2017). In this systematic review the studies investigating the internal KCFs with OpenSim or SIMM software used either the generic “gait2392” OpenSim model (Meireles et al., 2016) or equations by Winby et al. (2009), which are also based on Delp et al. (1990) Kumar et al. (2013); Winby et al. (2013); Manal et al. (2015); Saxby et al. (2016); Wellsandt et al. (2017); Khandha et al. (2019). We assume that similar coordinate system definitions were used. Additionally, a higher number of DoF at the knee joint was shown to overestimate the KCF because of an increased force in the quadriceps muscle. In contrast, more physiological constraints at the knee joint lead to an underestimation of KCF (Valente et al., 2015). Furthermore, models vary in muscle parameters as muscles' peak isometric force and affecting the calculation of muscle activation and forces during gait (Roelker et al., 2017). A previous study, which compared muscle force estimation between OpenSim and Anybody, reported that variations in muscle forces were mainly caused by dissimilar anatomical definitions, contrasts in calculated joint centers and segmental interactions of the models (Trinler et al., 2019). Further on, the calculation of joint contact forces and muscle forces appear to be more sensitive for changes in musculoskeletal definitions compared to joint angles and moments when varying body landmark positions, musculotendon geometry, or maximum muscle tension (Valente et al., 2014). Nevertheless, these changes only moderately affect model outcomes like joint contact forces and muscle forces. These aspects imply that a detailed description of the used MSK model is necessary and helpful for comparisons between studies.

Another parameter influencing the estimated outcome of the internal KCFs could be the implementation of participant-specific EMG data. Six studies (Kumar et al., 2013; Winby et al., 2013; Manal et al., 2015; Saxby et al., 2016; Wellsandt et al., 2017; Khandha et al., 2019) used a similar MSK model (Winby et al., 2009) and 4 of these studies (Kumar et al., 2013; Manal et al., 2015; Wellsandt et al., 2017; Khandha et al., 2019) implemented the EMG data from the same muscles. Two other studies (Winby et al., 2013; Saxby et al., 2016) used 1 respectively 3 additional EMG data from other muscles. Nevertheless, these 6 studies demonstrated similar results regarding the correlation between mKCF with KAM and KFM. Additionally, no substantially differences between studies using EMG-informed models compared to models without implementing participant-specific EMG data (Ogaya et al., 2014; Meireles et al., 2016; Richards et al., 2018) could be observed. However, other studies found a better correlation of estimated muscle forces, calculated with a best-fit solution taking muscle co-contraction into account with muscle forces measured with EMG compared to a static optimization approach (Martelli et al., 2015a). Therefore, we suggest that in case of movements with large muscle co-contraction, either EMG-driven musculoskeletal models or other approaches calculating the muscle forces are used.

Previous studies found that the different scaling approaches, e.g., body mass based scaling, scaling based on shape modeling or linear scaling affect the outcome of MSK modeling (Kainz et al., 2017; Bahl et al., 2019). In general, scaling based on medical images or with the inclusion of calculated joint centers into the scaling process improves the accuracy of the calculation of the hip joint center location compared to scaling with surface markers alone (Kainz et al., 2017; Bahl et al., 2019). However, the included studies in this systematic review performing MSK modeling only reported the usage of linear scaling based on marker positions and/or anatomical/anthropometrical data and no scaling based on medical images (Kumar et al., 2013; Winby et al., 2013; Ogaya et al., 2014; Giarmatzis et al., 2015, 2017; Manal et al., 2015; Wesseling et al., 2015; Meireles et al., 2016; Saxby et al., 2016; Wellsandt et al., 2017; Richards et al., 2018; Khandha et al., 2019). Moreover, the effect of soft tissue artifacts on MSK modeling should be considered as well. A previous study found a 5–25% variation of the joint moments and muscle forces and a relative variation of 5–15% of the joint contact forces when simulating soft tissue artifacts (Lamberto et al., 2017). Researchers should be aware of this aspect when interpreting gait analysis results with MSK modeling especially in cases with large soft tissue artifacts.

The studies using instrumented prosthesis to define the internal KCFs reported a good relationship between external knee joint moments and internal KCFs (Figure 3). The calculated external knee flexion-extension moments with the OpenSim model used from Kumar et al. (2013) and Winby et al. (2013) were previously validated with good reliability against data from an isokinetic dynanometer (Lloyd and Besier, 2003). In addition, the results obtained with another MSK model (Manal et al., 2015; Saxby et al., 2016; Wellsandt et al., 2017; Richards et al., 2018; Khandha et al., 2019) were already validated against directly measured internal KCFs of an instrumented prosthesis (Gerus et al., 2013; Manal and Buchanan, 2013; Lund et al., 2015), also with good agreement. Since the generic models were previously validated against different methods and have been used by a large number of researchers, we assume that the calculation of external joint moments and internal joint contact forces measured by MSK models is valid to assess the internal joint loading.

#### 4.2.4. Effect of Walking Speed

In former investigations it was reported that walking speed affects KAM and KCF but also HAM and HCF. A fast walking speed increases the first peak and decreases the second peak of KAM and HAM (Schwartz et al., 2008) whereas both peaks of tKCF (Lerner et al., 2014) and HCF (Giarmatzis et al., 2015) increase. Included studies reported walking speed between 1.1 m/s and 1.6 m/s while Trepczynski et al. (2014) and Richards et al. (2018) did not state any walking speed information. The effect of walking speed on the relationship between external knee joint moments and internal KCFs was investigated by Kutzner et al. (2013). They found an increased *R*^2^ but no significant *R*^2^-change when combining walking speed with first peak of the external KAM to predict mKCF. While an effect of walking speed on the external and internal joint loading parameter exists, we suggest to further study its influence on the relationship between external knee joint moments and internal KCFs.

## 5. Conclusion and General Recommendations

Seventeen studies have been found that analyzed a relationship between internal and external joint loading parameters. For the investigated populations, it can be summarized that the first peak or total maximal value of mKCF were best predicted by KAM alone and in combination with KFM. Additionally, the first peak of tKCF was well predictable by KAM. In contrast, the internal mKCF and tKCF in the second half of stance were only low correlated with the external knee joint moments. The internal *l*KCF also correlated only weakly with the external knee joint moments during the entire stance phase. The peak HCF in the first half of stance is strongly predictable by HAM, however, less strong at the second peak during the second half of stance. For the first half of stance, the determination of HAM is sufficient enough whereas statements about the second half of stance should be made by calculating the internal HCF.

The estimation of external joint moments is useful for a general statement about the mKCF or tKCF peak in the first half of stance or for a maximal loading. In addition, the calculation of external joint moments is implemented in most gait labs in the general processing procedures and therefore easily accessible. In contrast, MSK modeling is usually not part of the clinical assessment and therefore requires higher computational cost. Moreover, when evaluating joint contact forces from MSK modeling, output errors due to misaligned muscle point positions or marker based scaling should be taken into account. Nevertheless, investigating diseases like valgus malalignment of the lower limb, calculating the *l*KCF by MSK modeling should be preferred, or at least additionally consulted, because the external joint moments from ID do not correlate strongly with *l*KCF.

Altogether ID and MSK modeling are two different methods of analyzing joint loading. The method that should be used in the clinical environment depends on the clinical question, since for some applications computing external joint moments is sufficient, whereas a greater amount of time may be justified, e.g., for patients with valgus malalignment.

## Data Availability Statement

The original contributions presented in the study are included in the article/Supplementary Material, further inquiries can be directed to the corresponding author/s.

## Author's Note

All of the authors listed in the byline were fully involved in the study and preparation of the manuscript. They have made substantial contributions to the conception, design, execution, or interpretation of the reported study and fulfill the requirements for authorship established by the International Committee of Medical Journal Editors. Each of the authors has read and concurs with the content in the final manuscript.

## Author Contributions

JH and FS conceived the presented idea. JH and UT drafted the overview of the review and performed the analysis and interpretation. JH drafted the manuscript and visualized the results. UT, AM, and FS reviewed the manuscript, suggested improvements in the content and approved the final version. All authors agreed to be accountable for all aspects of the work.

## Conflict of Interest

The authors declare that the research was conducted in the absence of any commercial or financial relationships that could be construed as a potential conflict of interest.

## Abbreviations

ACLR: anterior cruciate ligament reconstruction
APM: arthroscopic partial meniscectomy
BMI: body mass index
BW: body weight
DoF: degree of freedom
EMG: electromyography
F_sup: superior force
HAM: hip adduction moment
HCF: hip joint contact force
HEM: hip extension moment
HFM: hip flexion moment
HRM: hip rotation moment
ID: inverse dynamics
KAM: knee adduction moment
KCF: knee joint contact force
KEM: knee extension moment
KFM: knee flexion moment
lKCF: lateral knee joint contact force
mKCF: medial knee joint contact force
mKOA: medial knee osteoarthritis
MSK: musculoskeletal
OA: osteoarthritis
RMSE: root mean squared error
tKCF: total knee joint contact force
TKR: total knee replacement.
