# Supplementary material for: A Systematic Review of the Associations Between Inverse Dynamics and Musculoskeletal Modeling to Investigate Joint Loading in a Clinical Environment
Source: Front Bioeng Biotechnol. 2020 Dec 7;8:603907. doi: 10.3389/fbioe.2020.603907 (PMC7750503; doi:10.3389/fbioe.2020.603907)
Supplement: Supplementary file 1 [file Data_Sheet_1.pdf]

A Systematic Review of the Associations Between Inverse Dynamics and Musculoskeletal Modeling to Investigate Joint Loading in a Clinical Environment

Supplementary Material

J. Holder [1,2,*], **U. Trinler** [3], **A. Meurer** [4] and **F. Stief** [1,2]

[1]Faculty of Medicine, Goethe University Frankfurt, Frankfurt/Main, Germany
[2]Movement Analysis Laboratory, Orthopedic University Hospital Friedrichsheim gGmbH, Frankfurt/Main, Germany
[3]Laboratory for Movement Analysis, BG Trauma Center Ludwigshafen, Ludwigshafen, Germany
[4]Department of Special Orthopedics, Orthopedic University Hospital Friedrichsheim gGmbH, Goethe University Frankfurt, Frankfurt/Main, Germany

Correspondence*:
J. Holder: Marienburgstr. 2, 60528 Frankfurt/Main, Germany
E-mail: j.holder@friedrichsheim.de

A FULL SEARCH TERMS

The search was performed on November 14, 2019.

- Pubmed:
  ((((gait OR walk*) AND (hip OR knee) AND (force* OR moment* OR torque*) AND ((model* OR muskuloskeletal OR musculoskeletal) OR (“inverse dynamics” OR simulation*)) AND ((full text[sb] AND hasabstract[text]) AND (“1990/01/01”[PDat] : “2019/10/31”[PDat]) AND Humans[Mesh] AND (English[lang] OR German[lang])))))
- Web of Science:
  (((TS=(gait OR walk*) AND TS=(hip OR knee)) AND TS=(force* OR moment* OR torque*)) AND TS=((model* OR muskuloskeletal OR musculoskeletal) OR (“inverse dynamics” OR simulation*))) AND TS = human*
  Databases= WOS, BIOABS, BCI, CCC, DRCI, DIIDW, KJD, MEDLINE, RSCI, SCIELO, ZOOREC
  Timespan=1990-2019
  Search language=English

B QUALITY ASSESSMENT CATEGORIES AND PARAMETERS

Table S1. Quality assessment categories and parameters adopted from Downs and Black (1998). A total score (in %) for every category and an overall total score (in %) are calculated for every study dividing the reached points per category with the maximal possible points in this category. The overall score of each paper was calculated by dividing the total reached points with the maximal possible points.

	Category	Parameter	Maximal points
1	Aims	Aim	2
		Patients & controls	2
2 & 3	Patients & Controls	Pathology	1
		Inclusion/exclusion criteria	2
		Quantity	4
		Gender	2
		Age & spread	4
		Body height, BW or BMI + spread	8
		Knee alignment	2
4	Equipment	Capture system	2
		Nr. of cameras	1
		Frequency cameras	1
		Nr. of markers	1
		Marker locations	1
		Filter (cameras)	2
		Force plates	1
		Nr. of force plates	1
		Frequency force plates	1
		Filter (force plates)	2
5	EMG	EMG system	1
		Frequency EMG	1
		Muscles	1
		Data processing	1

BW: body weight; BMI: body mass index; EMG: electromyography; DoF: degree of freedom.

Continued on next page

Table S1. *Continued from previous page*

	Category	Parameter	Maximal points
6	External joint moments/forces	Segments Nr.	1
		Segments name	1
		DoF	2
		Software (angles)	1
		Description of model (angles)	1
		Software (moments)	1
		Description of model (moments)	1
		Peak, mean, etc. (moments)	1
		Unit (moments)	1
7	Internal joint contact forces	Segments Nr.	1
		Segments name	1
		DoF (knee or hip)	1
		Software	1
		Description of model	1
		Scaling approach	1
		Optimization type	1
		Nr. of muscle-tendon units	1
		Peak, mean, etc.	1
		Unit	1
8	Statistics	Walkway or treadmill	1
		Walking speed + spread	2
		Shod/barefoot	1
		Software	1
		Clear description	2
		Statistical output	2
9	Discussion & conclusion	Discussion & conclusion	2
		Total points	75

BW: body weight; BMI: body mass index; EMG: electromyography; DoF: degree of freedom.

C STATISTICAL METHODS AND EXTRACTED PARAMETERS

Table S2. Statistical methods and full list of extracted parameters. *RMSE*: root mean squared error.

Study	Statistical method	Extracted parameters
**Knee joint**		
Esculier et al. (2017)	Linear regression	Unstandardized regression coefficients (*B*, standard error), *t*, *p*, cumulative *R*, cumulative adjusted R^2, significant *F* changes
Khandha et al. (2019)	Pearson correlation	*r*, *p*
Kumar et al. (2013)	(Multiple) linear regression	Unstandardized regression coefficients (*B*, standard error), β, *p*
Kutzner et al. (2013)	Linear regression	R^2, *p*
Manal et al. (2015)	Linear regression	Unstandardized regression coefficients (*B*, standard error), *t*, *p*, *r*, adjusted R^2, significant *F* changes
Meireles et al. (2016)	(Multiple) linear regression	R^2, *p*
Meyer et al. (2013)	Linear regression	R^2 and RMSE
Noyes et al. (1992)	Pearson correlation	*r*, *p*
Ogaya et al. (2014)	Pearson correlation	*r*, *p*
Richards et al. (2018)	Linear regression	Regression coefficients (*B*, standard error) and 95 % confidence intervals, *p*, adjusted R^2, RMSE, % error
Saxby et al. (2016)	General linear models	*p*, R^2, normalized RMSE
Trepczynski et al. (2014)	Linear mixed-effects models	Regression coefficients, RMSE (Across all activities: R^2, RMSE, *p*)
Wellsandt et al. (2017)	Hierarchical linear regression	R^2, *p*
Winby et al. (2013)	Pearson correlation	R^2, *p*
**Hip joint**		
Giarmatzis et al. (2017)	Stepwise regression	R^2, *p*
Giarmatzis et al. (2015)	Regression (linear mixed models)	R^2, *p*
Wesseling et al. (2015)	(Multiple) linear regression	R^2, *p*

D RELATIONSHIP BETWEEN THE HIP JOINT CONTACT FORCE AND EXTERNAL HIP JOINT MOMENTS

Table S3. Results for hip joint contact force: Relationship for the first and second peaks.

Study	Age [years]	Independent variable	Walking speed [m/s]	First peak		Second peak	
				R^2	p	R^2	p
Giarmatzis et al. (2017)	69.6	HAM	0.83	0.82	< 0.05		
			1.11	0.81	< 0.05		
			1.38	0.81	< 0.05		
			1.67	0.67	< 0.05	0.60	< 0.05
	21.4	HAM	0.83	0.65	< 0.05		
			1.11	0.55	< 0.05		
			1.38	0.55	< 0.05		
			1.67	0.67	< 0.05		
	69.6	HEM	0.83			0.57	< 0.05
			1.11			0.70	< 0.05
			1.38			0.46	< 0.05
		HEM + HAM + HRM	0.83			0.87	< 0.05
	21.4	HRM	0.83			0.40	< 0.05
			1.11			0.63	< 0.05
			1.38			0.66	< 0.05
			1.67			0.76	< 0.05

HAM: hip adduction moment; *HEM*: hip extension moment; *HRM*: hip rotation moment.

Continued on next page

Table S3. *Continued from previous page*

Study	Age	Independent variable	Walking speed [m/s]	First peak R^2	First peak p	Second peak R^2	Second peak p
Giarmatzis et al. (2015)	22.2	HAM	0.83	0.53	< 0.05	0.16	> 0.05
			1.11	0.53	< 0.05	0.17	> 0.05
			1.38	0.54	< 0.05	0.09	> 0.05
			1.67	0.78	< 0.05	0.12	> 0.05
		HEM	0.83	0.001	> 0.05	0.52	< 0.05
			1.11	0.04	> 0.05	0.51	< 0.05
			1.38	0.14	> 0.05	0.61	< 0.05
			1.67	0.17	> 0.05	0.7	< 0.05
		HRM	0.83	0.04	> 0.05	0.44	< 0.05
			1.11	0.02	> 0.05	0.03	> 0.05
			1.38	0.07	> 0.05	0.01	> 0.05
			1.67	0.02	> 0.05	0.12	> 0.05
		HAM + HEM	1.67	0.93	< 0.05		
		HFM + HRM	0.83			0.66	< 0.05
Wesseling et al. (2015)	52-61	HAM	1.28	0.87	< 0.001	0.54	< 0.001
		HFM		0.02	0.007	0.20	< 0.001
		HRM		0.85	< 0.001	0.32	< 0.001
		HAM + HRM		0.88	< 0.001	0.55	< 0.001
		HFM + HAM		0.89	< 0.001	0.76	< 0.001
		HRM + HFM		0.90	< 0.001	0.57	< 0.001
		HRM + HFM + HAM				0.76	< 0.001

HAM: hip adduction moment; *HEM*: hip extension moment; *HRM*: hip rotation moment.

E RELATIONSHIP BETWEEN JOINT MOMENTS AND THE MEDIAL KNEE JOINT CONTACT FORCE FOR THE FIRST AND SECOND PEAK

Table S4. Results for medial knee contact force: Relationship for the first and second peak.

Study	Population group	Independent variable	First peak R^2	First peak p	Second peak R^2	Second peak p
Kutzner et al. (2013)	Patients (TKR)	KAM	0.45	0.046	0.55	0.022
Richards et al. (2018)	Patients (mKOA)	KAM	0.597	< 0.05	0.439	< 0.05
Wellsandt et al. (2017)	Patients (ACLR) (involved leg)	KFM	0.072	0.150		
	Patients (ACLR) (uninvolved leg)		0.036	0.319		
Richards et al. (2018)	Patients (mKOA)	KAM + KFM	0.733	< 0.05	0.430	< 0.05
Wellsandt et al. (2017)	Patients (ACLR) (involved leg)	KFM + KAM	0.343	0.003		
	Patients (ACLR) (uninvolved leg)		0.396	0.001		
Kumar et al. (2013)*	Patients (mKOA) & controls	KAM	**0.74**	0.002	**0.13**	0.297
Winby et al. (2013)	Patients (APM) & controls	KAM	0.40	< 0.05		
Kumar et al. (2013)*	Patients (mKOA) & controls	KFM	**0.14**	0.047	**0.00**	0.896
Winby et al. (2013)	Patients (APM) & controls	KFM	0.25	< 0.05		
Ogaya et al. (2014)	Controls	KAM	**0.518**	< 0.001	**0.397**	< 0.001
		KFM	**0.240**	< 0.001	**0.012**	0.252
		KEM	**0.005**	0.483	**0.449**	< 0.001
Meyer et al. (2013)	Patients (TKR)	F_sup	0.380		0.162	
		F_sup + KAM	0.475		0.417	
		F_sup + KAM + KFM	0.480		0.418	

TKR: total knee replacement; *mKOA*: medial knee osteoarthritis; *ACLR*: anterior cruciate ligament reconstruction; *APM*: arthroscopic partial meniscectomy; *KAM*: knee adduction moment; *KFM*: knee flexion moment; *KEM*: knee extension moment; *F_sup*: superior force.

Values in **blue**: R^2 is calculated from original r-value.

* The authors performed a multiple linear regression analysis and reported β, a standardized regression coefficient which can be compared to r if only one independent variable is used in the multiple linear regression analysis.

F RELATIONSHIP BETWEEN JOINT MOMENTS AND THE MEDIAL KNEE JOINT CONTACT FORCE FOR THE TOTAL MAXIMAL VALUES

Table S5. Results for medial knee contact force: Relationship for the total maximal values.

Study	Population group	Independent variable	Total maximal value R^2	Total maximal value p
Khandha et al. (2019)	Patients (ACLR) (involved leg)	KAM	**0.608**	< 0.001
	Patients (ACLR) (uninvolved leg)		**0.348**	< 0.001
Manal et al. (2015)	Patients (ACLR)	KAM	0.633	0.004
		KAM + KFM	0.851	0.009
Noyes et al. (1992)	Patients (ACLR) & controls	KAM	**0.176**	< 0.05
		KFM	**0.090**	> 0.05
		KEM	**0.593**	< 0.01
Ogaya et al. (2014)	Controls	KAM	**0.348**	< 0.001
Esculier et al. (2017)	Controls (all)	KAM	0.314	< 0.001
	Females (only)		0.518	< 0.001
	Males (only)		0.518	< 0.001
Saxby et al. (2016)	Controls	KAM	0.36	< 0.05
Ogaya et al. (2014)	Controls	KFM	**0.068**	0.122
		KEM	**0.360**	< 0.001
Esculier et al. (2017)	Controls (all)	KAM + KFM	0.749	< 0.001
	Females (only)		0.925	< 0.001
	Males (only)		0.826	< 0.001

ACLR: anterior cruciate ligament reconstruction; *KAM*: knee adduction moment; *KFM*: knee flexion moment; *KEM*: knee extension moment.
Values in **blue**: R^2 is calculated from original r-value.

G RELATIONSHIP BETWEEN JOINT MOMENTS AND THE TOTAL KNEE JOINT CONTACT FORCE FOR THE FIRST AND SECOND PEAK

Table S6. Results for total knee contact force: Relationship for the first and second peaks.

Study	Population group	Independent variable	First peak R^2	First peak p	Second peak R^2	Second peak p
Meireles et al. (2016)	Patients (early mKOA)	KAM	0.686	< 0.01	0.000	> 0.05
	Patients (established mKOA)		0.737	< 0.01	0.050	> 0.05
Richards et al. (2018)	Patients (mKOA)	KAM	0.098	< 0.05	0.036	> 0.05
Meireles et al. (2016)	Patients (early mKOA)	KFM	0.621	< 0.01	0.545	< 0.01
	Patients (established mKOA)		0.382	< 0.01	0.068	> 0.05
	Patients (early mKOA)	KAM + KFM	0.912	< 0.01	0.654	< 0.01
	Patients (established mKOA)		0.912	< 0.01	0.202	> 0.05
Richards et al. (2018)	Patients (mKOA)	KAM + KFM	0.441	< 0.05	0.027	> 0.05
Meireles et al. (2016)	Controls	KAM	0.651	< 0.01	0.189	> 0.05
		KFM	0.206	< 0.05	0.255	< 0.05
		KAM + KFM	0.857	< 0.01	0.660	< 0.01
Saxby et al. (2016) (total maximal value)	Controls	KAM	0.15	< 0.05		
Meyer et al. (2013)	Patients (TKR)	F_sup	0.351		0.068	
		F_sup + KFM	0.575		0.069	

mKOA: medial knee osteoarthritis; *KAM*: knee adduction moment; *KFM*: knee flexion moment; *F_sup*: superior force.
